# Personalized hypertension management based on serial assessment and telemedicine (PHMA): a cluster randomize controlled trial protocol in Anhui, China

**DOI:** 10.1186/s12872-021-01943-5

**Published:** 2021-03-12

**Authors:** Xingrong Shen, Siyi Xiao, Rong Liu, Guixian Tong, Tongzhu Liu, Debin Wang

**Affiliations:** 1 School of Public Health, Anhui Medical University, 81 Meishan Road, Hefei, China; 2 The First Affiliated Hospital of USTC, 17 Lujiang Road, Hefei, China

**Keywords:** Cluster randomized controlled trial, Hypertension, Behavioral intervention, Personalized management, China

## Abstract

**Background:**

Despite tremendous investment worldwide, hypertension treatment and control rates remain low. The complexity and long-term dynamics of influencing factors make personalized management inevitable and challenging. This protocol describes Personalized Hypertension Management in Anhui, China (PHMA), a project that uses a package of innovative approaches in tailoring interventions to individual patient’s dynamic complications and contexts.

**Methods/design:**

PHMA strives to reduce hypertension harms by eight “objective behaviors” (e.g., self-monitoring and reporting, healthy diet, physical exercise/activities). These objective behaviors are promoted through five intervention measures: support for self- monitoring, supervised machine communications, daily education or reminder messages, weekly blood pressure notification, and quarterly signed feedback. PHMA uses ten categories and over 300 variables in selecting and refining intervention procedures and content for individual patients. Efficacy of the intervention package is evaluated using a cluster randomized controlled trial design involving a total of 60 site communities and 3352 hypertension patients. Primary measure for the evaluation is systolic and diastolic blood pressure; while secondary evaluation measures include quality of life (EQ5D-5L), occurrence of hypertension-related complications (such as cerebral hemorrhage, coronary heart disease, myocardial or cerebral infarction), healthcare utilization and scores of objective behaviors.

**Discussion:**

PHMA uses novel, low cost and sustainable approaches to tailor interventions to the dynamic conditions and contexts of individual patients. Unlike contemporary approaches to hypertension management which are mainly population based, each participant patient in PHMA applies a unique intervention package and all messages, feedbacks and other materials sent out to individual patients are different from each other. PHMA is the first project that adopts comprehensive tailoring and if proved effective, it should have important implications for future research, practice and policy-making.

*Trial registration* ISRCTN10999269. July 17, 2020; https://doi.org/10.1186/ISRCTN10999269.

**Supplementary Information:**

The online version contains supplementary material available at 10.1186/s12872-021-01943-5.

## Background

The number of patients with hypertension has reached 1.13 billion worldwide [1]. It has increased by 30.0% since 1990 and is projected to increase to 1.56 billion by 2025 [2]. Hypertension causes heavy disease and economic burden. It is the most important risk factor for stroke, heart disease, renal dysfunction and other major diseases [3] and causes an annual deaths of about 9.4 million (17.8% of the total deaths) globally [4]. Hypertension epidemic and burden in China are equally serious. Total hypertension patients and annual deaths attributable to the disease in the country amounted to 300 and 2.5 million respectively [5, 6]. The nation’s annual direct medical expenses due to cardiovascular diseases was estimated as 130 billion yuan (or 18.4 billion USD), of which 36.6 billion yuan (or 5.2 billion USD) was for hypertension treatment [7].

Human responses to hypertension can be divided into two main areas, i.e., clinical treatment and behavioral intervention. Clinical responses comprise antihypertension medication and detection and treatment of hypertension complications. Behavioral interventions include medication adherence and lifestyle modifications, e.g., healthy diet, physical exercise, alcohol and tobacco containment, stress and insomnia coping, and family engagement [8–12]. However, the effectiveness of existing efforts falls far from expected. Most published interventions demonstrated only marginal to moderate efficacy [13, 14]. According to systematic reviews published by The Lancet in 2019, the treatment and control rate in high-income countries was 60.6% and 36.8% respectively [3]; and 29.9% and 10.3% in low- and middle-income countries [15]. The treatment and control rate in China was 38.3% and 14.5% [16].

Although a variety of reasons are attributable to the low treatment and control rates, lack of adequate sensitivity to the real needs of individual patients may play a key role. Contemporary approaches are mainly population rather than individual oriented [17–19]. Population level initiatives are highly feasible since they use only a single protocol for all individuals but suffer from inherent inability in tailoring interventions to the heterogeneous and changing conditions and contexts of individual residents [20]. Both antihypertension treatment and behavior modifications belong to life-time endeavor needing strong persistence under complex and dynamic contexts. This complexity and long-term dynamics make personalized hypertension management (PHM) inevitable since the chance to find identical hypertension determinant systems from different patients decreases exponentially as the number of its influencing factors multiplied by the number of potential variations in each of the factors at different time points increases.

However, PHM requires: a sustained information mechanism capable of constantly collecting and preserving data about relevant status and trajectory of individual patients; a sensitive planning mechanism capable of identifying personalized needs from the serial and multisource data and designing tailored management accordingly; and an effective delivery mechanism capable of implementing the planned interventions on a patient-by-patient base. These are almost impractical with traditional or mainly manual hypertension management. Due to rapidly advancing computing and communication technologies, more and more hypertension patients are using electronic tonometer, electronic weight scale, smartphone and other mobile devices in monitoring their blood pressure (BP) and related conditions [21–23]. This protocol describes a novel PHM package in Anhui, China (hereafter referred to as PHMA) which takes advantages of these new development and incorporates practical technologies from mobile and telemedicine, cloud computing, and programmed algorithms and models in a synergetic way in tackling the PHM requirements.

## Aim/objectives

The primary goal of this study is to test the efficacy of PHMA. The study hypothesis is that hypertension patients in the intervention arm will, compared to those in the control condition, demonstrate: (a) lower systolic and diastolic BP: (b) higher scores on objective behaviors including self-monitoring, treatment adherence, healthy diet, physical activities, tobacco/alcohol containment, anxiety/insomnia coping, family engagement; and (c) reduced use of medical care due to hypertension and its complications. A second objective of this study is to identify key facilitators, barriers and corresponding strategies in disseminating and implementing PHMA.

## Methods/design

### Intervention ingredients and tailoring

#### Overall framework

**Fig. 1:**
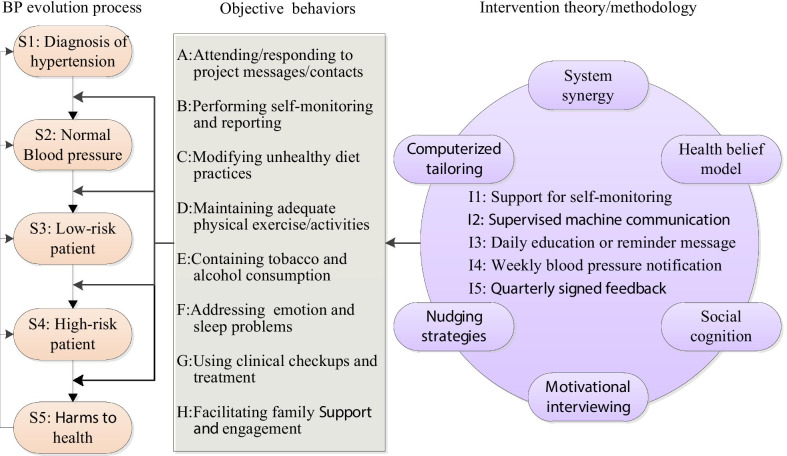
Conceptual framework of personalized management for hypertensive patients

As shown in Fig. 1, PHMA aims at preventing and reducing hypertension harms by eight objective behaviors including: (a) attending and responding to project messages/contacts; (b) performing self-monitoring and reporting; (c) modifying unhealthy diet habits or practices; (d) maintaining adequate physical exercise/activities; (e) containing tobacco and alcohol consumption; (f) addressing emotion and sleep problems; (g) using clinical checkups and treatment; and (h) facilitating family engagement. These objective behaviors are promoted through two intervention stages and five intervention measures. More specifically, the intervention for each participating patient starts with an orientation stage followed by a problem-solving stage. The orientation stage aims at equipping the patient with essential knowledge and attitudes about each of the objective behaviors applicable and helping him/her start to practice these behaviors. While the problem-solving stage facilitates the patient to identify and solve problems or barriers encountered in practicing the objective behaviors initiated in the orientation phase. The five intervention measures are: support for self-monitoring (I1), supervised machine communication (I2), daily education or reminder message (I3), weekly blood pressure notification (I4), and quarterly signed feedback (I5). Design of detailed content or procedures of these measures is guided by popular theories or strategies including system synergy [24], health belief model [25], social cognition theory [26], motivational interviewing [27], nudging strategies [28] and computerized tailoring. The following subsections briefly introduce each of the intervention measures.

#### Support for self-monitoring (I1)

**Fig. 2:**
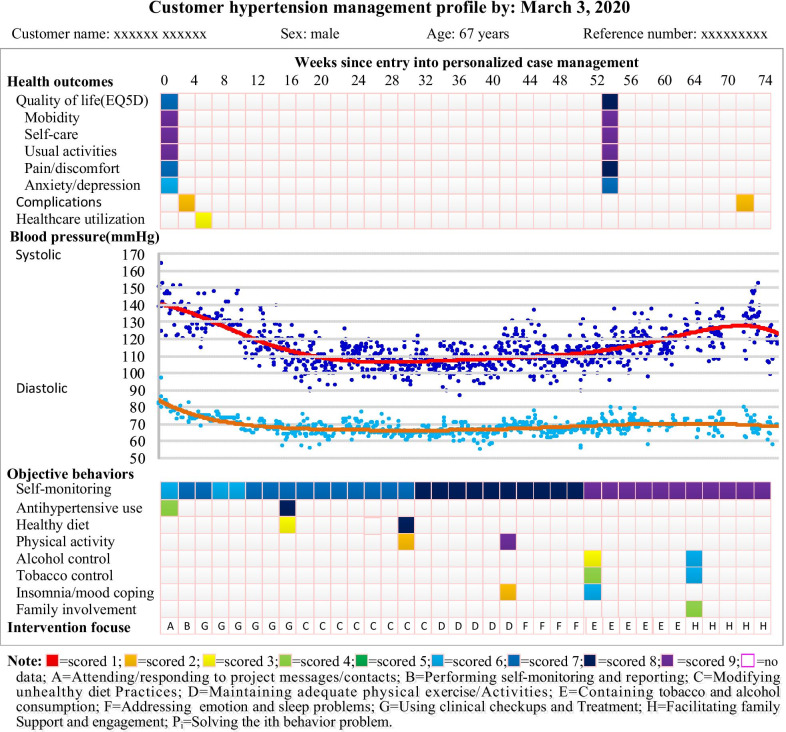
Example personalized hypertensive management profile

PHMA provides necessary technical support for participant patients to facilitate their practice of the objective behaviors especially self-monitoring. First, it maintains a cloud database and web-based support system capable of receiving, storing and processing patients’ self-monitored data and allowing them to view their personal records and feedbacks. Second, the project provides each participant patient with an electronic tonometer which: allows the patient to measure point SBP, DBP and pulse at home; sends the results to a cloud data center maintained by the project; and receives and plays voice messages disseminated from the cloud center. Third, the project encourages the participating patients to log onto their personal online accounts to: view their hypertension management profile (Fig. 2) and all the messages, notifications, feedbacks and other IEC (information, education and communication) materials he/she has received in the past; and administer relevant self-monitoring questionnaires (Additional file 2: Appendix 1) as suggested.

#### Supervised machine communication (I2)

PHMA delivers supervised machine communication (SMC) via telephone at the beginning when the patient enrolls into the project and then at the end of every three months. The communication is conducted mainly by an artificial intelligence (AI) voice processing system according to preset transcripts but the process is supervised by a human professional to handle exceptional machine-patient interactions. SMC comprises baseline and follow-up sessions lasting for about half an hour each time. The baseline SMC introduces PHMA and invites participation in the project, and, if successful, performs a brief yet comprehensive assessment of the patient’s: (a) diagnosis and treatment history; (b) symptoms of hypertension complications; and (c) BP-related lifestyle practices. Each of the follow-up SMC session focuses primarily on the patient’s hypertension management in the past 3-monthes and discusses: status and trends of his/her BP; efforts and progresses made in managing his/her hypertension; difficulties encountered; and plans or suggestions for overcoming the difficulties.

#### Daily education or reminder message (I3)

PHMA sends short (less than 120 Chinese characters), daily (once a day), and bi-modality (text and voice) messages via: (a) voice electronic tonometer; and (b) WeChat (the most popular social media in China), if the patient is a frequent WeChat user; or (c) mobile phone, if the patient uses mobile phone but are not used to WeChat. Content of the messages differs from patient to patient and from time to time depending on the actual sequence of objective behaviors to be promoted and behavior problems to be addressed. Taking the example shown in Fig. 2, the “intervention focus” during weeks 16–32 was “C” and so all messages send to that specific patient during these weeks centered on “modifying unhealthy diet”. All the messages are purposefully kept short to minimize burden of reading/ listening and a “continued one message every day” strategy is adopted to maintain a consistent bond with the patient and reinforce the objective behavior continuously.

#### Weekly blood pressure notification (I4)

**Fig. 3:**
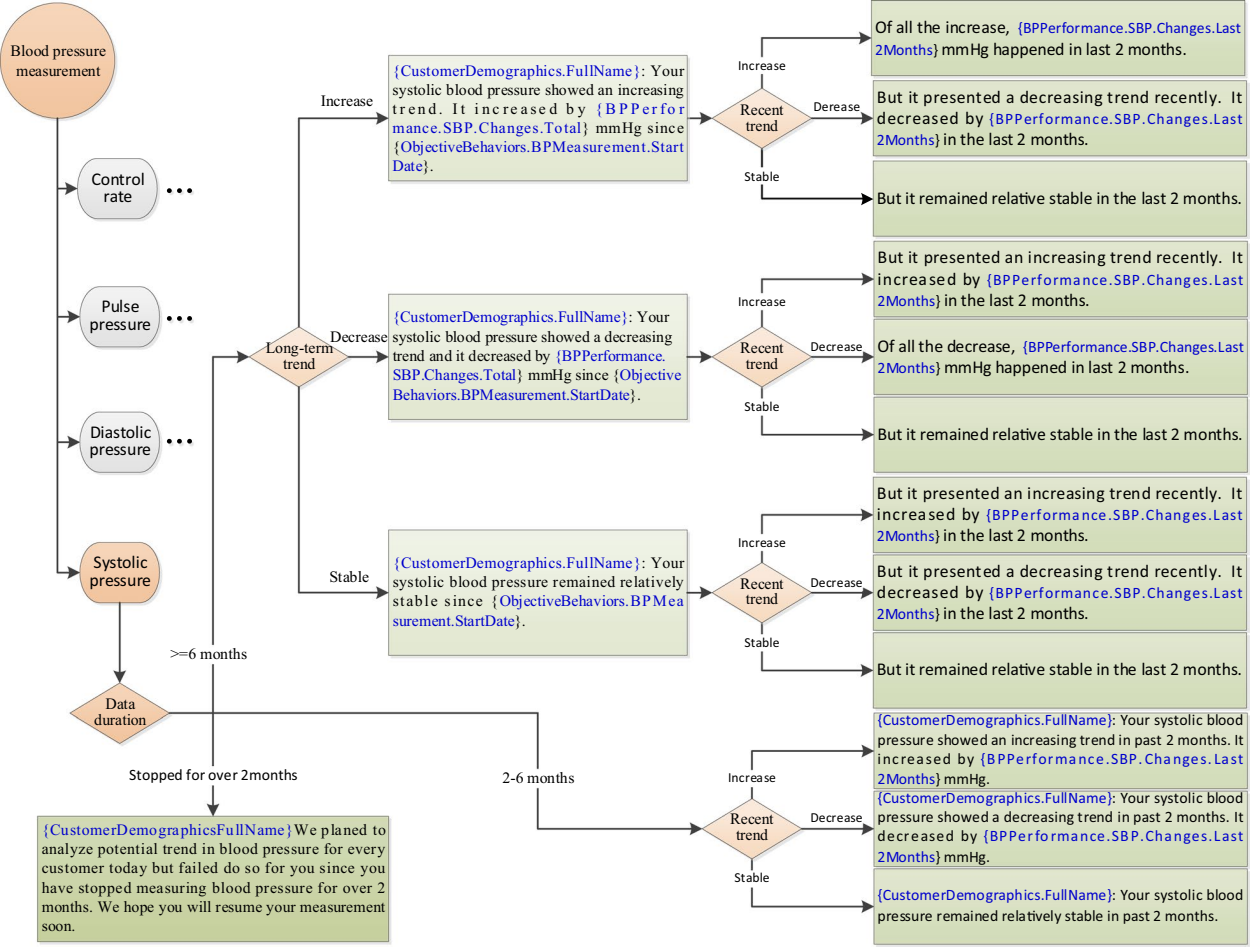
Example flowchart of how blood pressure notification is generated using patient self-measurements

PHMA sends a weekly notification about trends in SBP, DBP, pulse BP and BP control rate in turn for every participating patient. In other words, if a patient receives a short notification about his/her SBP at the end of this week, he/she will receive a notification about DBP, pulse BP and BP control rate at the end of the next one, two and three week(s) respectively. Then these are repeated for every four weeks. Figure 3 uses SBP as an example and illustrates how the content of the notification is generated using the patient’s self-measured blood pressure data.

#### Quarterly signed feedback (I5)

**Fig. 4:**
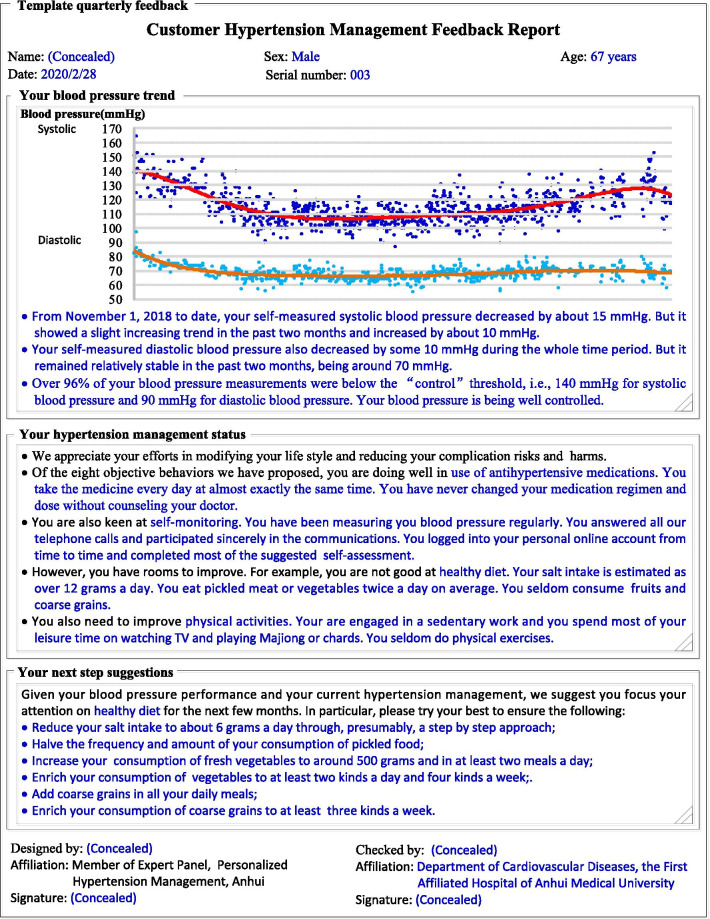
Example signed quarterly feedback (simplified to fit into one page)

PHMA disseminates quarterly feedbacks to each of its participant patients via: (a) paper letter sent via postal service; (b) personal webpage; and (c) WeChat. In the orientation phase, each feedback addresses a specific objective behavior; while in the implementing phase, each feedback addresses a specific barrier to the objective behaviors. All feedbacks comprises five parts: (a) identity and time, to indicate that the feedback is current and addressed to the specific patient named; (b) BP performance, to tell the patient about his/her BP status and trend and indicate that the feedback centers on his/her BP; (c) efforts made recently, to appreciate the patient’s efforts and progresses in the previous 3-months and identify main areas for improvement; (d) actions to take next, to propose feasible actions for the patient to take in the next period; and (e) signatures, to tell the patient that the feedback is produced and checked by authorized professionals and thus useful and reliable. Figure 4 provides an example feedback.

#### Methods for tailoring interventions

**Fig. 5:**
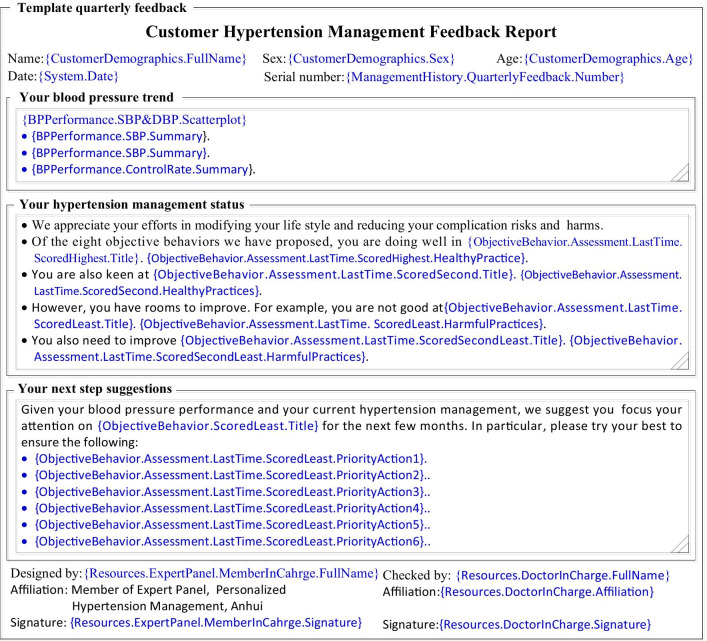
Template signed quarterly feedback

PHMA uses comprehensive approaches in tailoring interventions to the need and context of individual patients. First, PHMA requires that the intervention for any specific patient in any specific time period focuses on only one most important and feasible objective behavior or behavior problem determined by a priority score. This score is automatically generated by a computerized algorithm based on data accumulated from previous SMC with the patient and his/her self-monitored data. Second, each item in the IEC materials pool of PHMA is designed as a template inserted with variables to be replaced with relevant values, text or diagrams according to the actual conditions/contexts of the patient under concern. The following is a “template” message: *“{CustomerDemographics.FullName}: Capping monthly salt intake is an effective way in controlling blood pressure. You have {CustomerDemographics.FamilyMember. Number} members living together. So, your family should consume less than {ObjectiveBehavior.HealthyDiet.MonthlyFamilySalt} grams of salt a month in total.”* As indicated by “{}”, this message contains three variables. When sent to a patient named “Zhang San” who has 3 family members (two adults and a child of 8 years), the message is changed into: *“Zhang San: Capping monthly salt intake is an effective way in controlling blood pressure. You have 3 members living together. So, your family should consume less than 180 g of salt a month in total.”* Fig. 5 shows the template of the quarterly signed feedbacks mentioned earlier and Fig. 4 is an example feedback generated from this template.

#### Variables for characterizing patients

PHMA maintains a growing pool of variables for characterizing individual patients. All these variables are classified, coded and computerized in a way that they can be easily: (a) added to relevant IEC templates in intervention development; and (b) replaced, in intervention implementation, with relevant values, text or images according to available data about the specific patient under concern. By the time this paper is written, a total of ten categories and over 300 of variables had been computerized. Table 1 presents all the categories identified so far and example variables under each category.

**Table 1:** Categories and examples of variables used to tailor interventions

Demographics of patients (CustomerDemographics)
- Patient’s full name (CustomerDemographics. FullName);
- Patient’s Sex (CustomerDemographics.Sex);
- Patient’s Family name (CustomerDemographics. FamilyName);
……
Status and trend of blood pressure (BPPerformance)
- Overall mean of SBP (BPPerformance.SBP.Mean.Overall);
- Mean of SBP for the last 10 times (BPPerformance.SBP..Mean.Last10Times);
- Overall trend of DBP in past year (BPPerformance.DBP.Trend.LastYear);
……
Patient outcome (PatientOutcome)
- Score of quality of life (EQ5D) as assessed last time (PatientOutcome.QoL.LastAssessment.Score);
- Date when quality of life was last assessed (PatientOutcome.QoL LastAssessment.Date);
- Complications occurred in the past year (PatientOutcome.Complications.LastYear. All);
……
Clinical history in relation to hypertension (ClinicalHistory)
- Year in which hypertension was diagnosed (ClinicalHistory.Hypertension.Diagnosis.Year);
- Whether medication treatment is in use (ClinicalHistory.Hypertension.Treatment.Current.Status);
- Type of current medications (ClinicalHistory.Hypertension.Treatment.Current.Type);
……
Objective behaviors (ObjectiveBehaviors)
- Title of the objective behavior scored the highest in previous assessment (ObjectiveBehaviors.LatestAssessment. ScoredHighest.Title);
- Score of “healthy diet” in the latest assessment (ObjectiveBehaviors.LatestAssessmen.HealthyDiet.Score);
- Title of objective behavior scored the least in latest assessment (ObjectiveBehaviors.LatestAssessment. ScoredLeast.Title);
……
Problems with or barriers to objective behaviors (BehaviourProblems)
- List of problems or barriers identified from the latest assessment (BehaviourProblems.LatestAssessment.FullList);
- Title of problem scored the highest in the latest assessment (BehaviourProblems.LatestAssessment. ScoredHighest.Title); - Description of problem scored the highest in the latest assessment (BehaviourProblems.LatestAssessment. ScoredHighest.Description); ……
Hypertension-related physical indicators (PhysicalIndicator)
- Body weight as measured last time (PhysicalIndicator.BodyWeight.LastMeasurement.Value);
- Date when patient body was last weighted (PhysicalIndicator.BodyWeight.LastMeasurement.Date);
- Waist circumference as measured last time (PhysicalIndicator.WeistCircumference.LastMeasurement.Value);
……
Daily activities (DailyActivity)
- Common working activities as reported last time (DailyActivity.LastReport.Work.Description);
- Common working hours as reported last time (DailyActivity.LastReport.Work.Hours);
- Common leisure activities as reported last time (DailyActivity.LastReport.Leisure.Description);;
……
Social relations (SocialRelations)
- Number of family members (SocialRelations.FamilyMember.Number);
- Member of family members (SocialRelations.FamilyMember.RelationList);
- Family member in closest contact with the patient (SocialRelations.FamilyMember.InClosestContact.Relations);
……
Resources relating to hypertension management (Resources)
- Family income per year (Resources.Faimly.AnnualIncome)
- Minutes needed to get to the nearest hypertension clinic (Resources.MinutesToNearestClinic);
- Name of doctor in charge of the patient (Resources.DoctorInCharge.Current.FullName);
……

“()” defines the start and end of the code of the variable or variable category before it

### Study design and settings

The study adopts a cluster randomized controlled trial (RCT) design involving a total of 60 site communities with 12 in the control arm and 48 in the intervention arm. The control arm maintains existing hypertension case management; while the intervention arms, personalized hypertension management as described above. Project evaluation applies to both arms using the same data collection methods and by same field data collectors. The uneven distribution of site communities between the control and intervention arms was designed to enable detection of potential differences between the control arm and at least four main subgroups with different intervention ingredients within the intervention arm. The analysis and reporting of the trial will strictly follow the CONSORT guidelines [29, 30]. A Trial Steering Committee has been established which holds annual meetings and supervises the trial implementation. For time schedule of the trial please refer to Additional file 1.

### Selection and randomization of communities

The RCT is implemented in Anhui, an inland province located in eastern China with a population of about 70 million living in 105 cities or counties. Selection of participating sites and patients uses a clustered randomization which proceeds in the following steps: step1 divides Anhui province into north, middle and south regions; step 2 randomly selects 4 cities/counties from each of the three regions; step 3 randomly selects five non-adjacent communities from each of the cities/counties selected; step 4 randomly selects 56 patients diagnosed with hypertension from the communities selected. The five communities selected from each city/county in steps 3 are randomly assigned to the control arm (n = 1) and intervention arm (n = 4). Hypertensive patients aged 18 years or above and capable and willing to participate in the study from the selected communities meet the inclusion criteria. All the randomization is performed by a statistical professional from outside the project team. The study will be blinded to both data collectors and analyst.

### Withdrawal

Any participant can withdraw from the study at any time. The participants withdrawn from the study will not be replaced but the withdrawal reasons will be recorded.

### Study sample size

The above sample size of participating patients is calculated on base of the primary intervention assessment measures, i.e., changes in SBP. Based on our previous study results and our aim of comparing the effects of at least four combinations of main intervention ingredients, we suppose: (a) SBP reduction was about 5.0 mmHg as compared between the intervention and control arms; (b) standard deviation of the SBP reduction was 7.8 mmHg; (c) ICC value was 0.05; and (d) patients in the intention arm need to be assigned to four subgroups to receive four different combinations of interventions. So, to detect a possible absolute difference of 5.0 mmHg with 90% power and alpha 0.05, we need 228 patients in each arm. By allowing for a 20% attrition rate and a design effect of 2.45, the total sample size is estimated as 3352 (= 2.45 * 228 * 5 * 1.2) and this translates into 56 patients per community.

## Measures

The primary measures for assessing the efficacy of PHMA are SBP/DBP. The secondary measures include quality of life (EQ5D-5L), hypertension-related complications, healthcare utilization and objective behaviors.

### Data collection and management

All the above measures together with data about potential confounding variables (e.g., age, sex, education, years since hypertension diagnosis) are collected at the patients’ households by trained data collectors at baseline and 12 and 24 months after baseline. SBP/DBP is measured using a mercury sphygmomanometer in accordance with standard operation specifications [31]; while the remaining data are solicited using structured questionnaires (Additional file 2) administered face-to-face by the field data collectors. Data collected at the household will be double entered using EPI DATA V3.1.

Study documentation will be kept in locked files in the offices of the project assessment supervisor and the Principal Investigator. All data will be backed-up on separate media and stored in a secure filing cabinet. Access to security passwords will be given only to the Principal Investigator and the Assessment Supervisor. Personal identifiers will not be stored in the data set and all computers will be protected by antivirus software. These data safety and confidentiality procedures are overseen by a Data Monitoring Committee.

### Data analysis

The data collected will be used to compare the differences between the control groups and the intervention group as a whole and subgroups with different intervention ingredients in the intervention arm at different time points in terms of: (a) SBP/DBP; and (b) the secondary measures including quality of life, occurrence of hypertension-related complications, healthcare utilization, and scores of objective behaviors. Estimation of statistical significance and confidence intervals will assume a type I error established in alpha = 0.05, using the IBM SPSS V22 statistics package. Despite our anticipated high follow-up rates, we expect some extent of missing data, typically participants missing an interview and are then found at the next follow-up assessment. We will use appropriate statistical techniques to estimate the missing values and then perform the analysis on the completed sample.

Initial data analysis will consist of descriptive summaries intended to examine the patterns of the various measurements and check for normality of the continuous variables. Necessary transformations will be explored and selected, if necessary, to induce approximate normality. Regarding the numerical variables between two groups, t-test of independent samples for mean comparisons will be carried out. We will also employ generalized hierarchical linear models. These models will include multiple linear regression, as well as binary, ordinal, or nominal logistic regression. In these multilevel analyses, patient-specific measurements are included in the models.

### Adverse events

In this study, the most likely anticipated incident is the loss of privacy, so the identification information of patients will not be placed on response forms but use an unique reference number instead.

### Dissemination

Findings from the study will be summarized for the benefit of public health policymakers, health professionals and health service managers in China and elsewhere. A final workshop will be held for the purpose of presenting the evidence generated by this study and working with local and regional stakeholders to translate this evidence into public health action.

## Discussion

PHMA introduces innovative approaches to tailor interventions to the complex and dynamic BP and related complications and contexts of individual patients. Contemporary approaches to hypertension management are mainly population-based though some studies have tried some extent of differentiated interventions, e.g., send a reminder to patients who have failed to attend an appointment [32]. PHMA uses ten categories of variables in selecting and refining intervention procedures and content for individual patients. As a result, each participant patient in PHMA applies a unique intervention package and all messages, feedbacks and other materials sent out to individual patients are different from each other. To our knowledge, PHMA is the first project that adopts so comprehensive tailoring and if proved effective, it should have important implications for future research, practice and policy-making.

PHMA adopts pragmatic strategies in securing feasibility and containing cost of interventions. It uses multiple modalities (e.g., text and sound) and venues (e.g., telephone, WeChat, Webpages, paper leaflets) in forming and disseminating intervention materials to suit various patients, especially old and illiterate ones. It strives to leverage mobile devices, self-monitoring, distant communications and computerized programs into effective, low cost, convenient and sustainable interventions. Taking the example of SMC, it may lead to a novel and cost-effective mode of interactions between patients and health professionals. Although a human professional is required to supervise the process of every SMC session, his/her workload is reduced to a large extent as compared with traditional patient-doctor communications. In a traditional communication, the doctor (or other human professional) needs to complete a whole range of activities, e.g., reviewing the patient’s status and history, forming an outline plan, and interacting with the patient; while in a SMC session, what the doctor needs to do is only to start the conversation by a brief introduction and then listen to the machine-patient interactions and add occasional queries or comments. In addition to workload reduction for the doctor, SMC may also exhibit good quality and experience for the patient, since the procedures and content are preset by an expert panel and then refined by computerized algorithms according to the actual conditions of the patient under concern.

PHMA incorporates guidance from multiple theories. As illustrated in Fig. 1, design of detailed intervention content or procedures is guided by health belief model, social cognition theory, motivational interviewing, nudging strategies and system synergy. All these theories/strategies have been used successfully in a variety of populations and settings [33–35]. Theory-informed intervention design prevents omission of important aspects and thus enhances overall efficacy [34, 36]. Each of the theories used in PHMA has its own strengths: health belief model is useful in addressing behaviors driven by rational thinking; nudging strategies apply to behaviors triggered by automatic cognitive processes [25, 28]; motivational interviewing is keen at generating and maintaining adequate momentum to leverage sustained behavior changes; system synergy informs incorporation of all intervention ingredients in a way that prevents equifinality and maximizes cost-effectiveness [27, 35, 37].

## Supplementary Information

**Additional file 1.** Schedule of enrolment, interventions, and assessments of participants.

**Additional file 2.** Selected patient assessment questionnaires and temporary scoring systems.

**Additional file 3.** Patient Consent Record & Contact Details.

## Data Availability

Not applicable.

## Abbreviations

PHMA: Personalized hypertension management
SMC: Supervised machine communication
BP: Blood pressure
SBP: Systolic blood pressure
DBP: Diastolic blood pressure
IEC: Information, education and communication
RCT: Randomized controlled trial
