# Supplementary material for: Personalized hypertension management based on serial assessment and telemedicine (PHMA): a cluster randomize controlled trial protocol in Anhui, China
Source: BMC Cardiovasc Disord. 2021 Mar 12;21:135. doi: 10.1186/s12872-021-01943-5 (PMC7953659; doi:10.1186/s12872-021-01943-5)
Supplement: Supplementary file 1 — Additional file 1. Schedule of enrolment, interventions, and assessments of participants. [file 12872_2021_1943_MOESM1_ESM.docx]

Additional file 1 The schedule of enrolment, interventions, and assessments of participants

| **Activities** | **Time point** | | | | | | | | | | | |
| --- | --- | --- | --- | --- | --- | --- | --- | --- | --- | --- | --- | --- |
|  | **Year 1** | | | | **Year 2** | | | | **Year3** | | | |
|  | Q1 | Q2 | Q3 | Q4 | Q1 | Q2 | Q3 | Q4 | Q1 | Q2 | Q3 | Q4 |
| **Enrollment** |  |  |  |  |  |  |  |  |  |  |  |  |
| *eligibility screen* | ✓ |  |  |  |  |  |  |  |  |  |  |  |
| *informed consent* | ✓ |  |  |  |  |  |  |  |  |  |  |  |
| *allocation* |  |  | ✓ |  |  |  |  |  |  |  |  |  |
| **Intervention** |  |  |  |  |  |  |  |  |  |  |  |  |
| *I1-support for self-monitoring* |  |  |  | ✓ | ✓ | ✓ | ✓ | ✓ | ✓ | ✓ | ✓ |  |
| *I2-supervised machine communication* |  |  |  | ✓ | ✓ | ✓ | ✓ | ✓ | ✓ | ✓ | ✓ |  |
| *I3-daily education or reminder message* |  |  |  | ✓ | ✓ | ✓ | ✓ | ✓ | ✓ | ✓ | ✓ |  |
| *I4-weekly blood pressure notification* |  |  |  | ✓ | ✓ | ✓ | ✓ | ✓ | ✓ | ✓ | ✓ |  |
| *I5-quarterly signed feedback* |  |  |  | ✓ | ✓ | ✓ | ✓ | ✓ | ✓ | ✓ | ✓ |  |
| **Assessment** |  |  |  | ✓ |  |  |  | ✓ |  |  |  | ✓ |
