# Supplementary material for: Personalized hypertension management based on serial assessment and telemedicine (PHMA): a cluster randomize controlled trial protocol in Anhui, China
Source: BMC Cardiovasc Disord. 2021 Mar 12;21:135. doi: 10.1186/s12872-021-01943-5 (PMC7953659; doi:10.1186/s12872-021-01943-5)
Supplement: Supplementary file 2 — Additional file 2. Selected patient assessment questionnaires and temporary scoring systems. [file 12872_2021_1943_MOESM2_ESM.docx]

# Appendix 1 Selected patient assessment questionnaires and temporary scoring systems

## Part A: Health outcomes

### A1: Quality of life

Overall quality of life (a1) is scored by TTO (time trade off) value between different conditions defined by the following five dimensions.

A1a (mobility): What would you say about your mobility? (Please tick **one** item that suits you best)

[ ] You have no problems in walking about (a1a=5)

[ ] You have slight problems in walking about (a1a=4)

[ ] You have moderate problems in walking about (a1a=3)

[ ] You have severe problems in walking about (a1a=2)

[ ] You are unable to walk about? (a1a=1)

A1b (self-care): What would you say about your self-care? (Please tick **one** item that suits you best)

[ ] You have no problems washing or dressing yourself (a1b=5)

[ ] You have slight problems washing or dressing yourself (a1b=4)

[ ] You have moderate problems washing or dressing yourself (a1b=3)

[ ] You have severe problems washing or dressing yourself (a1b=2)

[ ] You are unable to wash or dress yourself? (a1b=1)

A1c (usual activities): What would you say about your usual activities, for example work, study, housework, family or leisure activities? (Please tick **one** item that suits you best)

[ ] You have no problems doing your usual activities (a1c=5)

[ ] You have slight problems doing your usual activities (a1c=4)

[ ] You have moderate problems doing your usual activities (a1c=3)

[ ] You have severe problems doing your usual activities (a1c=2)

[ ] You are unable to do your usual activities (a1c=1)

A1d (pain/discomfort): What would you say about your pain or discomfort? (Please tick **one** item that suits you best)

[ ] You have no pain or discomfort (a1d=5)

[ ] You have slight pain or discomfort(a1d=4)

[ ] You have moderate pain or discomfort(a1d=3)

[ ] You have severe pain or discomfort(a1d=2)

[ ] You have extreme pain or discomfort(a1d=1)

A1e (anxiety/depression): What would you say about your anxiety or depression? (Please tick **one** item that suits you best)

[ ] You are not anxious or depressed(a1e=5)

[ ] You are slightly anxious or depressed(a1e=4)

[ ] You are moderately anxious or depressed(a1e=3)

[ ] You are severely anxious or depressed(a1e=2)

[ ] You are extremely anxious or depressed(a1e=1)

### A2: Complications

Overall score of complications equals sum of all the values assigned to the following items ticked and a2 is set as 9 if the sum is over 9.

A2a: Have you felt or experienced the following symptoms during the past month? (Please tick **all** items that suit you)

[ ] Headache or dizziness (a2a1=1)

[ ] Dazzling lights or black out (a2a2=1)

[ ] Tinnitus or syrigmus (a2a3=1)

[ ] Heartache or chest ache (a2a4=1)

[ ] Palpitation, (a2a5=1)

[ ] Memory loss (a2a6=1)

[ ] Inattention (a2a7=1)

[ ] insomnia or dreaminess (a2a8=1)

[ ] Easily excited and fidgety (a2a9=1)

[ ] Limb numbness (a2a10=1)

[ ] Edema of eyelid (a2a11=1)

[ ] Edema of lower extremities (a2a12=1)

A2b (hypertension related diagnosis): Have you ever been diagnosed by a doctor the following? (Please tick **all** items that suit you)

[ ] Coronary heart disease (a2b1=3)

[ ] Myocardial infarction (a2b2=3)

[ ] Cerebral infarction (a2b3=3)

[ ] Atherosclerosis (a2b4=3)

[ ] Impaired renal function (a2b5=3)

[ ] Hyperglycemia or diabetes (a2b6=2)

[ ] Hyperlipidemia (a2b7=2)

### A3: Healthcare utilization

Overall score of a3 equals sum of all the values assigned to the following items ticked and a3 is set as 9 if the sum is over 9.

A3a: Have you sought help from a doctor during the past month because of hypertension or hypertension-related health problems?

[ ] Yes (a3a=1)

[ ] No (a3a=0 and end of a3)

A3b: How many times have you sought help from a doctor during the past month because of hypertension or hypertension-related health problems?

[ ] One (a3b=0)

[ ] Two (a3b=1)

[ ] Three (a3b=2)

[ ] Four (a3b=3)

[ ] Five or more (a3b=4)

A3c: Have you been hospitalized during the past month because of hypertension or hypertension- related health problems?

[ ] Yes (a3c=2)

[ ] No (a3c=0 and end of a3)

A3d: What kinds of hospitals have you been hospitalized during the past month because of hypertension or hypertension- related health problems? (Please tick all that apply to you)

[ ] Township level hospital (a3d1=1)

[ ] County level hospital (a3d2=2)

[ ] Prefecture level hospital (a3d3=3)

[ ] Province or higher level hospital (a3d4=4)

A3e: How many days have you been hospitalized during the past month because of hypertension or hypertension- related health problems?

[ ] 3 days or less (a3e1=1)

[ ] 4-6 days (a3e2=2)

[ ] 7-9 days (a3e3=3)

[ ] 10-12 days (a3e3=4)

[ ] 13 days or longer (a3e5=5)

## Part B: Objective behaviors

### B1: Self-monitoring

Overall score of b1 for a patient is automatically generated on base of his/her self-monitored data on other modules (but without asking any questions here) and according to the following algorithms. B1 is set as 9 if the sum is over 9.

B1a: How many times has the patient monitored his/her blood pressure during the past two weeks?

[ ] 0 times (b1a=0)

[ ] 1-2 times ((b1a=1)

[ ] 3-4 times ((b1a=2)

[ ] 5 times or more ((b1a=3)

B1b: How many times has the patient monitored his/her health outcomes during the past year?

[ ] 0 times (b1b=0)

[ ] 1 times ((b1b=1)

[ ] 2 times ((b1b=2)

[ ] 3 times or more ((b1b=3)

B1c: How many times has the patient monitored his/her complications during the past year?

[ ] 0 times (b1c=0)

[ ] 1 times ((b1c=1)

[ ] 2 times ((b1c=2)

[ ] 3 times or more ((b1c=3)

B1d: How many times has the patient monitored his/her healthcare utilization during the past year?

[ ] 0 times (b1d=0)

[ ] 1 times ((b1d=1)

[ ] 2 times ((b1d=2)

[ ] 3 times or more ((b1d=3)

B1e: How many times has the patient monitored his/her antihypertensive use during the past year?

[ ] 0 times (b1e=0)

[ ] 1 times ((b1e=1)

[ ] 2 times ((b1e=2)

[ ] 3 times or more ((b1e=3)

B1f: How many times has the patient monitored his/her diet practice during the past year?

[ ] 0 times (b1f=0)

[ ] 1 times ((b1f=1)

[ ] 2 times ((b1f=2)

[ ] 3 times or more ((b1f=3)

B1g: How many times has the patient monitored his/her physical activity during the past year?

[ ] 0 times (b1g=0)

[ ] 1 times ((b1g=1)

[ ] 2 times ((b1g=2)

[ ] 3 times or more ((b1g=3)

B1h: How many times has the patient monitored his/her alcohol/tobacco consumption during the past year?

[ ] 0 times (b1h=0)

[ ] 1 times ((b1h=1)

[ ] 2 times ((b1h=2)

[ ] 3 times or more ((b1h=3)

B1i: How many times has the patient monitored his/her stress/mood coping during the past year?

[ ] 0 times (b1i=0)

[ ] 1 times ((b1i=1)

[ ] 2 times ((b1i=2)

[ ] 3 times or more ((b1i=3)

B1j: How many times has the patient monitored his/her family involvement during the past year?

[ ] 0 times (b1j=0)

[ ] 1 times ((b1j=1)

[ ] 2 times ((b1j=2)

[ ] 3 times or more ((b1j=3)

B1k: How many times has the patient monitored his/her body weight during the past year?

[ ] 0 times (b1k=0)

[ ] 1 times ((b1k=1)

[ ] 2 times ((b1k=2)

[ ] 3 times or more ((b1k=3)

### B2: Medication adherence

Overall score of b2 equals sum of all the values assigned to the following items ticked and b2 is set as 9 if the sum is over 9.

B2a: Have you had antihypertension medicine during the past month?

[ ] Yes (b2a=1)

[ ] No (b2a=0 or not applicable, and end of b2)

B2b: Have you forgotten to take antihypertension medicine during the past month?

[ ] Yes (b2b=0)

[ ] No (b2b=1)

B2c: Have you delayed or stopped to take antihypertension medicine because you were busy or out of home during the past month?

[ ] Yes (b2c=0)

[ ] No (b2c=1)

B2d: Have you delayed or stopped to take antihypertension medicine because you were ill during the past month?

[ ] Yes (b2d=0)

[ ] No (b2d=1)

B2e: Have you delayed or stopped to take antihypertension medicine because you were unhappy with your self during the past month?

[ ] Yes (b2e=0)

[ ] No (b2e=1)

B2f: Have you delayed or stopped to take antihypertension medicine because you were upset by others during the past month?

[ ] Yes (b2f=0)

[ ] No (b2f=1)

B2g: Have you reduced or increased the dose of antihypertension medicine you take on your own (without consult a doctor) during the past month?

[ ] Yes (b2g=0)

[ ] No (b2g=1)

B2h: Have you changed the kind of antihypertension medicine you take on your own (without consult a doctor) during the past month?

[ ] Yes (b2h=0)

[ ] No (b2h=1)

B2h: Please input the time (e.g., 18:30) when you first took antihypertension medicine on the past three days?

[ ] Yesterday[___:___]

(b2h1=0 if time difference between b2h1 and b2h3 >30 minutes; otherwise 1)

[ ] The day before yesterday[___:___]

(b2h2=0 if time difference between b2h2 and b2h1 >30 minutes; otherwise 1)

[ ] Three days ago[___:___]

(b2h3=0 if time difference between b2h3 and b2h2 >30 minutes; otherwise 1)

### B3: Healthy diet

Overall score of b3 equals sum of all the values assigned to the following items ticked and b3 is set as 9 if the sum is over 9.

B3a: To what extent does the following statement describe your salt intake in the past month?

B3a1: You took less than 6 grams of salt per day

[ ] Not at all (b3a1=0)

[ ] Partly (b3a1=1)

[ ] Definitely (b3a1=2)

B3a2: You seldom ate pickled food

[ ] Not at all (b3a2=0)

[ ] Partly (b3a2=1)

[ ] Definitely (b3a2=2)

B3a3: You seldom ate salty cookies, melon seeds etc.

[ ] Not at all (b3a3=0)

[ ] Partly (b3a3=1)

[ ] Definitely (b3a3=2)

B3b: To what extent does the following describe your vegetable consumption in the past month?

B3b1: You ate at least 400grams of fresh vegetables a day

[ ] Not at all (b3b1=0)

[ ] Partly (b3b1=1)

[ ] Definitely (b3b1=2)

B3b2: At least 2 out of your 3 daily meals contained fresh vegetables

[ ] Not at all (b3b2=0)

[ ] Partly (b3b2=1)

[ ] Definitely (b3b2=2)

B3b3: You ate at least 4 different kinds of fresh vegetables a week

[ ] Not at all (b3b3=0)

[ ] Partly (b3b3=1)

[ ] Definitely (b3b3=2)

B3b4: You seldom ate leftover vegetables from previous meals

[ ] Not at all (b3b4=0)

[ ] Partly (b3b4=1)

[ ] Definitely (b3b4=2)

B3c: To what extent does the following describe your fruit consumption in the past month?

B3c1: You ate at least 400grams of fresh fruits a day

[ ] Not at all (b3c1=0)

[ ] Partly (b3c1=1)

[ ] Definitely (b3c1=2)

B3c2: You ate fresh vegetables at least twice a day

[ ] Not at all (b3c2=0)

[ ] Partly (b3c2=1)

[ ] Definitely (b3c2=2)

B3c3: You ate at least 4 different kinds of fresh fruits a week

[ ] Not at all (b3c3=0)

[ ] Partly (b3c3=1)

[ ] Definitely (b3c3=2)

B3c4: You seldom made juice out of whole fruits before consumption

[ ] Not at all (b3c4=0)

[ ] Partly (b3c4=1)

[ ] Definitely (b3c4=2)

B3d: To what extent does the following describe your whole grain consumption in the past month?

B3d1: At least 2 out of your 3 daily meals contained whole gains

[ ] Not at all (b3d1=0)

[ ] Partly (b3d1=1)

[ ] Definitely (b3d1=2)

B3d2: You ate at least 3 different kinds of whole grains a week

[ ] Not at all (b3d2=0)

[ ] Partly (b3d2=1)

[ ] Definitely (b3d2=2)

B3e: To what extent does the following describe your consumption of refined or “main” food or easily digestible and energy rich food in the past month?

B3e1: Each of your 3 meals a day contained less than half (in volume) of refined food

[ ] Not at all (b3e1=0)

[ ] Partly (b3e1=1)

[ ] Definitely (b3e1=2)

B3e2: When having meals, you always ate whole grains before refined food

[ ] Not at all (b3e2=0)

[ ] Partly (b3e2=1)

[ ] Definitely (b3e2=2)

B3e3: You seldom ate cookies, cakes etc. before, after and between meals

[ ] Not at all (b3e3=0)

[ ] Partly (b3e3=1)

[ ] Definitely (b3e3=2)

B3e4: You seldom put sugar in your meals or drinks

[ ] Not at all (b3e4=0)

[ ] Partly (b3e4=1)

[ ] Definitely (b3e4=2)

B3f: To what extent does the following describe your consumption of meat and fat in the past month?

B3f1: You seldom had more than 100 grams of red meat in a meal

[ ] Not at all (b3f1=0)

[ ] Partly (b3f1=1)

[ ] Definitely (b3f1=2)

B3f2: You seldom had meat with visible fat in your meals

[ ] Not at all (b3f2=0)

[ ] Partly (b3f2=1)

[ ] Definitely (b3f2=2)

B3f3: You seldom use animal oil but vegetable oil as cooking oil

[ ] Not at all (b3f3=0)

[ ] Partly (b3f3=1)

[ ] Definitely (b3f3=2)

### B4: Physical activity

Overall score of b4 equals sum of all the values assigned to the following items ticked and b4 is set as 9 if the sum is over 9.

B4a: To what extent does the following statement describe your leisure time exercise in the past month?

B4a1: You exercised for at least 5 days a week

[ ] Not at all (b4a1=0)

[ ] Partly (b4a1=1)

[ ] Definitely (b4a1=2)

B4a2: You exercised for at least 30 minutes every time

[ ] Not at all (b4a2=0)

[ ] Partly (b4a2=1)

[ ] Definitely (b4a2=2)

B4a3: Your leisure time exercise was seldom canceled by bad weather, travel or other unexpected events.

[ ] Not at all (b4a3=0)

[ ] Partly (b4a3=1)

[ ] Definitely (b4a3=2)

B4b: To what extent does the following statement describe your efforts in fitting physical activities with your daily working and living schedules in the past month?

B4b1: Whenever possible, you chose to walk or ride instead of driving or use of elevator, public transportation etc.

[ ] Not at all (b4b1=0)

[ ] Partly (b4b1=1)

[ ] Definitely (b4b1=2)

B4b2: Whenever possible, you chose to more energy consuming ways to complish your daily tasks instead of less energy consuming ones.

[ ] Not at all (b4b2=0)

[ ] Partly (b4b2=1)

[ ] Definitely (b4b2=2)

B4b3: You purposefully added additional energy consuming tasks into your daily working and living schedules, e.g., regular gardening, workplace visits.

[ ] Not at all (b4b3=0)

[ ] Partly (b4b3=1)

[ ] Definitely (b4b3=2)

B4c: To what extent does the following statement describe your efforts in reducing sedentary time in the past month?

B4c1: You regularly reviewed your daily activities in order to find out your sedentary time

[ ] Not at all (b4c1=0)

[ ] Partly (b4c1=1)

[ ] Definitely (b4c1=2)

B4c2: Whenever possible, you changed your sedentary way of working or entertainment into standing or walking ways.

[ ] Not at all (b4c2=0)

[ ] Partly (b4c2=1)

[ ] Definitely (b4c2=2)

B4c3: You purposefully inserted alternative activities into all your sedentary working or entertainment that lasts over 1 hour.

[ ] Not at all (b4c3=0)

[ ] Partly (b4c3=1)

[ ] Definitely (b4c3=2)

B4d: To what extent does the following statement describe your efforts in exercising your joints in the past month?

B4d1: You exercised all your joints every day when wake up

[ ] Not at all (b4d1=0)

[ ] Partly (b4d1=1)

[ ] Definitely (b4d1=2)

B4d2: You stretched or turned your arms, legs, neck, waist etc. whenever you engaged in long-time sedentary work or entertainment.

[ ] Not at all (b4d2=0)

[ ] Partly (b4d2=1)

[ ] Definitely (b4d2=2)

B4e: How would you describe the level of your physical activities in the past month?

[ ] very light (b4e=0)

[ ] light (b4e =1)

[ ] heavy (b4e =2)

[ ] very heavy (b4e=3)

B4f: How long was your total sedentary time (e.g., 2:30) in the past three days?

[ ] Yesterday[___:___]

[ ] The day before yesterday[___:___]

[ ] Three days ago[___:___]

(b4f=0, 1, 2 or 3, if average sedentary time was over 3, 2 to 3, 1 to 2 and less than 1 hours)

### B5: Alcohol/tobacco control

Overall score of b5 equals sum of all the values assigned to the following items ticked and b5 is set as 9 if the sum is over 9.

B5a: Have you consumed any alcohol in the past month?

[ ] Yes (b5a=0)

[ ] No (b5a=4 and skip to b5b)

B5a1: How frequently did you drink alcohol in the past month?

[ ] less than 3 times a week(b5a1=3)

[ ] 4 to 7 times a week(b5a1=2)

[ ] 8 to 14 times a week(b5a1=1)

[ ] 15 times or more a week(b5a1=0)

B5a2: Have you tried to reduce alcohol consumption in the past month?

[ ] Yes, very much (b5a2=0)

[ ] Yes, to some extent (b5a2=1)

[ ] No (b5a2=2 and skip to b5b)

B5b: Have you smoked in the past month in the path month?

[ ] Yes (b5b=0)

[ ] No (b5b =4 and skip to b5c)

B5b1: How many cigarettes did you smoke per day in the past month?

[ ] less than 5 cigarette a day(b5b1=3)

[ ] 5 to 10 cigarette a day (b5b1=2)

[ ] 11 to 20 cigarette a day (b5b1=1)

[ ] 21 cigarette or more a day k(b5b1=0)

B5b2: Have you tried to reduce cigarette smoking in the last month?

[ ] Yes, very much (b5b2=0)

[ ] Yes, to some extent (b5b2=1)

[ ] No (b5b2=2 and skip to b5c)

B5c: Have you been smoked in the past month in the path month?

[ ] Yes (b5c=0)

[ ] No (b5c =4 and end b5)

B5c1: How many times have been exposed to second hand smoking in the past month?

[ ] less than 3 times a week (b5c1=3)

[ ] 4 to 7 times a week (b5c1=2)

[ ] 8 to 14 times a week (b5c1=1)

[ ] 15 times or more a week (b5c1=0)

B5c2: Have you tried to avoid exposure to second hand smoking in the last month?

[ ] Yes, very much (b5c2=0)

[ ] Yes, to some extent (b5b2=1)

[ ] No (b5c2=2)

### B6: Insomnia/mood coping

Overall score of b6 equals sum of all the values assigned to the following items ticked and b6 is set as 9 if the sum is over 9.

B6a: Sleep quality as assessed using the Pittsburgh sleep quality index (PSQI)

[ ] PSQI =0-3(b6a=3)

[ ] PSQI =4-7(b6a=2)

[ ] PSQI =8-11(b6a=1)

[ ] PSQI =12-14(b6a=0)

B6b: Anxiety score as assessed using the Self-rating Anxiety Scale (SAS)

[ ] SAS<50(b6b=3)

[ ] SAS=50-59(6b=2)

[ ] SAS=60-69(b6b=1)

[ ] SAS>69(b6b=0)

B6c: Depression score as assessed using the Self-rating Depression Scale (SDS)

[ ]SDS=0-16(b6c=3)

[ ]SDS=17-34(b6c=2)

[ ]SDS=35-52(b6c=1)

[ ]SDS>52(b6c=0)

B6d: To what extent does the following statement describe your sleep-related activities in the past month?

B6d1: You kept your sleep time within 6 to 9 hours a day

[ ] Not at all (b6d1=0)

[ ] Partly (b6d1=1)

[ ] Definitely (b6d1=2)

B6d2: The main part of your daily sleep time was arranged at night

[ ] Not at all (b6d2=0)

[ ] Partly (b6d2=1)

[ ] Definitely (b6d2=2)

B6d3: You went to bed at a fixed hour every day

[ ] Not at all (b6d3=0)

[ ] Partly (b6d3=1)

[ ] Definitely (b6d3=2)

B6d4: You purposefully avoided certain activities like smoking, drinking tea or alcohol that may affect your sleep before your habitual sleep time every day

[ ] Not at all (b6d4=0)

[ ] Partly (b6d4=1)

[ ] Definitely (b6d4=2)

B6d5: You maintained a good sleeping environment with comfort bed, bedclothes, pillows, temperature, air and free from voice, noise, light and other disturbance.

[ ] Not at all (b6d5=0)

[ ] Partly (b6d5=1)

[ ] Definitely (b6d5=2)

B6e: To what extent does the following statement describe your practice coping with stress, anxiety and other mood problems in the past month?

B6e1: When encountered an unpleasant thing/feeling, you used to diver your attention to others

[ ] Not at all (b6e1=0)

[ ] Partly (b6e1=1)

[ ] Definitely (b6e1=2)

[ ] Not applicable (b6e1=2)

B6e2: When encountered an unpleasant thing/feeling, you used to analyze and understand the underline reasons

[ ] Not at all (b6e2=0)

[ ] Partly (b6e2=1)

[ ] Definitely (b6e2=2)

[ ] Not applicable (b6e2=2)

B6e3: When encountered an unpleasant thing/feeling, you used to analyze and understand it from different perspectives or view points

[ ] Not at all (b6e3=0)

[ ] Partly (b6e3=1)

[ ] Definitely (b6e3=2)

[ ] Not applicable (b6e3=2)

B6e4: You used to keep a diary of pleasant and meaningful things

[ ] Not at all (b6e4=0)

[ ] Partly (b6e4=1)

[ ] Definitely (b6e4=2)

B6e5: When encountered an unpleasant thing/feeling, you used to take relaxing actions, e.g., walking, playing table tennis, listening to musics.

[ ] Not at all (b6e5=0)

[ ] Partly (b6e5=1)

[ ] Definitely (b6e5=2)

[ ] Not applicable (b6e5=2)

B6e6: When encountered an unpleasant thing/feeling, you used to talk to your close friends or relatives.

[ ] Not at all (b6e6=0)

[ ] Partly (b6e6=1)

[ ] Definitely (b6e6=2)

[ ] Not applicable (b6e6=2)

B6e7: You used to anticipate and avoid potential events or social contacts that may lead to unpleasant things/feelings.

[ ] Not at all (b6e7=0)

[ ] Partly (b6e7=1)

[ ] Definitely (b6e7=2)

[ ] Not applicable (b6e7=2)

B6e8: You purposefully pursued certain activities that prevent or reduce unpleasant things/feelings.

[ ] Not at all (b6e8=0)

[ ] Partly (b6e8=1)

[ ] Definitely (b6e8=2)

[ ] Not applicable (b6e8=2)

### B7: Family engagement

Overall score of b7 equals sum of all the values assigned to the following items ticked and b7 is set as 9 if the sum is over 9.

B7a: Have you talked about your hypertension to any of family members of relatives in the past month?

[ ] Yes(b7a=1)

[ ] No(b7a=0)

B7b: Has any of your family members or relatives urged, reminded or provided help of any type and in any way for you to monitor your blood pressure in the past month?

[ ] Yes(b7b=1)

[ ] No(b7a=0)

B7c: Has any of your family members or relatives urged, reminded or provided help of any type and in any way for you to adhere to antihypertensive medications in the past month?

[ ] Yes(b7c=1)

[ ] No(b7c=0)

B7d: Has any of your family members or relatives urged, reminded or provided help of any type and in any way for you to contain salt intake in the past month?

[ ] Yes(b7d=1)

[ ] No(b7d=0)

B7e: Has any of your family members or relatives urged, reminded or provided help of any type and in any way for you to increase intake of fresh vegetables in the past month?

[ ] Yes(b7e=1)

[ ] No(b7e=0)

B7f: Has any of your family members or relatives urged, reminded or provided help of any type and in any way for you to increase intake of fruits in the past month?

[ ] Yes(b7f=1)

[ ] No(b7f=0)

B7g: Has any of your family members or relatives urged, reminded or provided help of any type and in any way for you to increase intake of whole grains in the past month?

[ ] Yes(b7g=1)

[ ] No(b7g=0)

B7h: Has any of your family members or relatives urged, reminded or provided help of any type and in any way for you to reduce intake of refined food or fat in the past month?

[ ] Yes(b7h=1)

[ ] No(b7h=0)

B7i: Has any of your family members or relatives urged, reminded or provided help of any type and in any way for you to increase physical activity or exercise in the past month?

[ ] Yes(b7i=1)

[ ] No(b7i=0)

B7j: Has any of your family members or relatives urged, reminded or provided help of any type and in any way for you to contain cigarette smoking in the past month?

[ ] Yes(b7j=1)

[ ] No(b7j=0)

B7k: Has any of your family members or relatives urged, reminded or provided help of any type and in any way for you to contain alcohol intake in the past month?

[ ] Yes(b7k=1)

[ ] No(b7k=0)

B7l: Has any of your family members or relatives provided help of any type and in any way for you to solve sleep problems in the past month?

[ ] Yes(b7l=1)

[ ] No(b7l=0)

B7m: Has any of your family members or relatives provided help of any type and in any way for you to cope with unpleasant mood in the past month?

[ ] Yes(b7m=1)

[ ] No(b7m=0)
