# Supplementary material for: Personalized hypertension management based on serial assessment and telemedicine (PHMA): a cluster randomize controlled trial protocol in Anhui, China
Source: BMC Cardiovasc Disord. 2021 Mar 12;21:135. doi: 10.1186/s12872-021-01943-5 (PMC7953659; doi:10.1186/s12872-021-01943-5)
Supplement: Supplementary file 3 — Additional file 3. Patient Consent Record & Contact Details. [file 12872_2021_1943_MOESM3_ESM.docx]

**Personalized hypertension management study**

**Patient Consent Record & Contact Details**

Dear patient

We invite you to participate in the project named “Development of personalized hypertension case management based on home blood measurement and human-machine joint counseling data” funded by the National Natural Science Foundation of China. This study has been approved by the Ethics Committee of Anhui Medical University. In order to protect your rights, please read the following statement of consent.

**Consent statement (and see overleaf)**

| 1. I understand what this study is about and how I am being asked to participate in it. |  |
| --- | --- |
| 1. I understand that taking part in this research is voluntary and that I am free to leave the study at any time, without giving any reason, and without my medical care or rights or those of my family being affected. |  |
| 1. I understand that any information I provide will be anonymised by removing all identifying details and will be kept strictly confidential and used only for research purposes. After the study, the anonymised information will be made publicly available for potential further research, but it will not be possible to identify me from the data. |  |
| 1. I agree to be contacted for an supervised machine communication. |  |
| 1. I understand that with my agreement, the interview will be audio-recorded but no one except the research team will hear this recording. I am aware that anonymised quotes from the interview may be included in reports of the study findings. |  |
| 1. My consent is based on the Anhui Medical University complying with their duties and obligations under the Data Protection Act. |  |

I understand the above statements and am fully aware of the risks and benefits that may be incurred in this study. I voluntarily participate in this study.

**Participant ID:** ___________________________

**Participant Contact Details**

Address: ______________________________

Phone number1: _______________________

Phone number2: _______________________

**Participant Consent (when the participant is not able to sign for themselves, the researcher will sign on their behalf to record their consent):**

|  |  |  |  |  |
| --- | --- | --- | --- | --- |
| Name of Participant |  | Date |  | Signature |
|  |  |  |  |  |
|  |  |  |  |  |
| Name of Family member |  | Date |  | Signature |
|  |  |  |  |  |
|  |  |  |  |  |
| Name of Person taking consent |  | Date |  | Signature |

If the patient refuses the above informed consent statement, ask why he/she chooses not to participate. Reason for refusal: ______________________________________________

**This information is to be either given to patients or discussed with the patients:**

Your involvement in the study will remain confidential and your study data will be anonymised. Your information will be given an identification number and any information that could identify you will be removed so it will not be possible to identify you in any way.

At the end of the study all the data will be made “Open Access” which means that it will be stored in an online database so that it is publicly available for future research. This information will only be available to other researchers and national bodies which monitor research studies and there will be no way to identify you from it.
